# A Nano-SiO_2_-Based Core-Shell Hybrid as a Dual-Functional Viscosity Reducer and Pour Point Depressant for Heavy Oil

**DOI:** 10.3390/polym18111295

**Published:** 2026-05-25

**Authors:** Borui Ji, Shuo Wang, Bauyrzhan Sarsenbekuly, Zhen Tao, Lijie Qi, Wanli Kang, Weiyu Duan, Hongbin Yang, Bo Zhang

**Affiliations:** 1 School of Energy and Petroleum Industry, Kazakh-British Technical University, Almaty 050000, Kazakhstan; jbr0108@163.com (B.J.); tao349562421@126.com (Z.T.); qilijie58@126.com (L.Q.); 2 Liaoning Inspection, Examination & Certification Centre, Key Laboratory of Testing and Quality Control for Petroleum Products, State Administration for Market Regulation, Shenyang 110032, China; sekiwangedward@163.com (S.W.);; 3 School of Petroleum Engineering, China University of Petroleum (East China), Qingdao 266580, China; hongbinyang@upc.edu.cn; 4 Shiyan Key Laboratory of Biological Resources and Eco-Environmental Protection, College of Chemical and Environmental Engineering, Hanjiang Normal University, Shiyan 442000, China; zhangbo2505@163.com

**Keywords:** heavy oil, viscosity reducer, pour point depressant, organic-inorganic hybrid, nano-SiO_2_

## Abstract

Heavy oil production and transportation are often restricted by high viscosity, poor mobility, and unfavorable low-temperature flow behavior, especially in waxy systems. While conventional polymer-based additives improve flow, they suffer from inadequate thermal stability, poor dispersibility in complex crude oil matrices, and insufficient multifunctionality. To address these issues, a nano-SiO_2_-based organic-inorganic hybrid flow improver, denoted as NSDA, was synthesized via in situ free-radical copolymerization of styrene, docosyl methacrylate, acrylic acid, and acrylamide on 3-(trimethoxysilyl)propyl methacrylate (KH-570)-modified silica surfaces. Characterization revealed that this core-shell nanohybrid structure significantly improved thermal stability and oil-phase dispersibility, maintaining nanoscale dispersion in xylene. A remarkable viscosity reduction rate of 90.2% was achieved, accompanied by a substantial pour point depression of 11 °C using only 0.5 wt% of NSDA in Liaohe heavy oil. This dual-functional performance is mainly attributed to the combined effects of the robust nano-SiO_2_ core and the multifunctional polymer shell, Specifically, the performance is driven by synergistic wax crystal regulation at low temperatures, alongside weakened intermolecular associations among polar heavy components and nanoparticle-assisted dispersion that govern viscosity reduction.

## 1. Introduction

Heavy oil resources constitute an important part of the global petroleum supply; however, their efficient production and transportation remain technically challenging because of their high viscosity, limited mobility, and complex chemical composition [1,2,3]. The rheological behavior of heavy petroleum systems is also strongly influenced by the relative contents of saturates, aromatics, resins, and asphaltenes, and may differ substantially from that of wax-dominated crude oils or bitumen-rich materials [4,5,6,7]. These interactions can promote the development of associated microstructures or supramolecular organizations in the oil phase, thereby hindering flow [8,9,10]. In waxy heavy oil systems, the situation becomes even more severe at low temperatures, where the crystallization and aggregation of paraffinic components can sharply increase the apparent viscosity and raise the pour point [11,12,13]. Accordingly, the development of efficient chemical agents capable of simultaneously reducing viscosity and improving low-temperature flowability is of considerable practical importance for heavy oil recovery and pipeline transportation.

Among the currently available technologies, chemical viscosity reduction based on polymer additives has received sustained attention because of its operational simplicity, rapid response, and compatibility with existing production processes [14,15]. A variety of polymeric materials, including styrene-maleic anhydride copolymers, hyperbranched polymers, star-shaped polymers, and comb-like methacrylate derivatives, have been investigated as viscosity reducers or pour point depressants for heavy and waxy oils. These materials can improve crude oil flow behavior by promoting the solubilization of heavy fractions, modifying wax crystallization behavior, and weakening intermolecular association within the oil matrix [16,17,18,19]. Nevertheless, conventional polymer systems still face several practical limitations, especially under harsh reservoir conditions. These limitations include inadequate thermal resistance, susceptibility to shear degradation, poor dispersibility in complex oil systems, and insufficient multifunctionality in addressing the coupled issues of heavy-component aggregation and wax deposition [20,21]. Therefore, there remains a clear need for new material designs that can combine structural robustness with multiple interfacial regulation functions.

In recent years, nanoparticle-assisted flow improvers have emerged as a promising strategy for heavy oil viscosity reduction. Because of their small size, high specific surface area, and tunable surface chemistry, nanoparticles can participate in the regulation of interfacial interactions in crude oil systems and, in some cases, provide a “micro-bearing” effect that contributes to improved mobility [22,23,24]. However, unmodified inorganic nanoparticles generally tend to self-aggregate and often show limited compatibility with the organic oil phase, which may restrict their effectiveness in complex heavy oil systems. To address these shortcomings, the construction of organic-inorganic hybrid materials has attracted increasing interest [25,26]. By grafting functional polymer chains onto nanoparticle surfaces, it becomes possible to combine the structural robustness of inorganic cores with the interfacial activity and molecular design flexibility of polymers, thereby improving dispersion stability and enabling synergistic performance [27].

From the standpoint of molecular design, the selection and combination of functional monomers play a decisive role in the performance of hybrid viscosity reducers. Long-chain alkyl methacrylates can co-crystallize with paraffinic waxes and interfere with the regular growth of wax crystals, thereby contributing to pour point depression and improved low-temperature fluidity [11,28]. Aromatic units such as styrene may strengthen interactions with aromatic heavy fractions and help regulate asphaltene aggregation through competitive π-related interactions [4,29]. In addition, polar monomers such as acrylic acid (AA) and acrylamide (AM) can introduce hydrogen-bonding and adsorption sites, which may weaken strong intermolecular association among polar components and improve the overall dispersion of heavy fractions [30]. The rational integration of these structural motifs into a single hybrid architecture may therefore offer an effective route to addressing multiple flow assurance problems in heavy oil simultaneously.

Based on these considerations, this study reports the synthesis of a nano-SiO_2_-supported organic-inorganic hybrid viscosity reducer, denoted as NSDA. Using 3-(trimethoxysilyl)propyl methacrylate (KH-570) as an interfacial coupling bridge [26], styrene (St), docosyl methacrylate (BMC), acrylic acid (AA), and acrylamide (AM) were graft-copolymerized in situ onto the surface of nano-SiO_2_ to construct a stable core-shell nanohybrid. In this design, nano-SiO_2_ serves as a rigid inorganic core to enhance thermal stability and provide nanoscale mechanical assistance, whereas the polymer shell integrates long alkyl chains, aromatic groups, and polar segments to achieve multifunctional interfacial regulation [31]. Compared with conventional oil-soluble polymer additives and previously reported nanoparticle-assisted flow improvers, the intended advancement of this work lies in three aspects. First, a covalently integrated core-shell architecture was constructed rather than a simple physical blend, which is expected to improve structural robustness and dispersion stability in an oil-like medium. Second, the monomer combination was designed to simultaneously address wax crystallization and the associative network of polar heavy components, thereby targeting both viscosity reduction and pour-point depression within one material. Third, the present study provides a structure-performance discussion that links the hybrid architecture, surface chemistry, and dispersion characteristics with the observed macroscopic flow-improvement behavior in a representative waxy heavy oil.

The structure, morphology, surface composition, thermal behavior, and oil-phase dispersibility of the obtained material were systematically characterized. Its viscosity-reduction and pour-point-depression performances were further evaluated using Liaohe heavy oil. Notably, experimental results revealed that at a minimal dosage of 0.5 wt%, NSDA achieved an impressive viscosity reduction rate of 90.2% and depressed the pour point by 11 °C. On this basis, the relationship between molecular/structural design and macroscopic performance is discussed, demonstrating that the excellent dual-functional performance is driven by the synergistic roles of solubilization, dispersion, wax inhibition, disruption of intermolecular interactions, and nanoparticle-assisted flow improvement. Ultimately, this work provides a practical material design strategy for multifunctional heavy oil flow improvers suitable for demanding reservoir environments.

## 2. Materials and Methods

### 2.1. Materials

The reagents used include styrene (St, AR), docosyl methacrylate (BMC, AR), xylene (AR), 3-(trimethoxysilyl)propyl methacrylate (KH-570, AR), tetraethyl orthosilicate (TEOS, AR), acrylamide (AM, AR), acrylic acid (AA, AR), 2,2’-azobisisobutyronitrile (AIBN, ≥98%), ammonia solution (NH_3_·H_2_O, ACS) and anhydrous ethanol (C_2_H_5_OH, AR). These were sourced from Aladdin Biochemical Technology Co., Ltd. (Shanghai, China). The main physicochemical information of the key reagents, including purity grade and representative molecular characteristics, is summarized in Table 1 according to the supplier’s technical data sheets. Deionized water prepared in the laboratory was used as the solvent. All chemicals were used as received without further purification.

### 2.2. Physicochemical Properties of the Heavy Oil Sample

**Figure 1:**
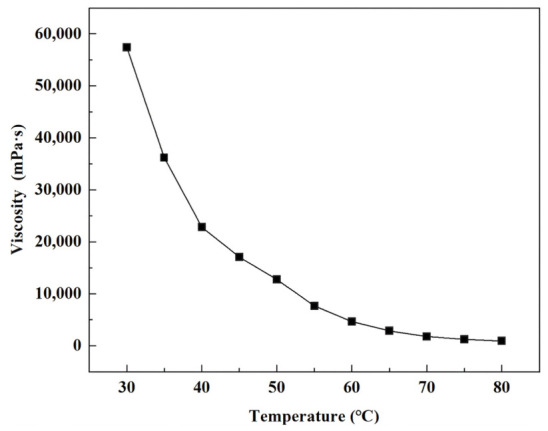
Temperature dependence of the viscosity of the heavy oil sample.

The heavy oil used in this study was obtained from the Liaohe Oilfield (Panjin, China). As shown in Figure 1, the viscosity of the oil decreased markedly with increasing temperature, indicating pronounced temperature-dependent flow behavior. At 50 °C, the apparent viscosity was 12,730 mPa·s, confirming the highly viscous nature of the sample.

The basic physicochemical properties and SARA composition of the heavy oil are summarized in Table 2. The SARA fractionation, including saturates, aromatics, resins, and asphaltenes, was carried out according to the Chinese petroleum industry standard NB/SH/T 0509-2010 [32]. The results show that the sample contained relatively high levels of resins (13.92 wt%) and asphaltenes (19.03 wt%), together with a wax content of 8.0 wt% and a pour point of 30 °C. These characteristics indicate that the oil can be classified as a typical high-viscosity heavy oil with poor low-temperature flowability [33,34]. The relatively high wax content and elevated pour point suggest a strong tendency toward wax crystallization, whereas the substantial resin and asphaltene contents are likely to contribute to strong intermolecular association and the development of viscosity-enhancing microstructures in the oil phase.

### 2.3. Preparation of NSDA

#### 2.3.1. Preparation of KH-570-Modified Nano-SiO_2_

Modified silica nanoparticles were prepared via a sol-gel process followed by surface functionalization. Briefly, ethanol (7.50 g), deionized water (2.30 g), and aqueous ammonia (0.23 g) were added to a 100 mL round-bottom flask and stirred at 30 °C for 10 min. An ethanolic solution of TEOS (10.00 g) was then added dropwise, and the mixture was heated to 80 °C and maintained for 1 h to generate the nano-SiO_2_ sol. After the system was cooled to 50 °C, an ethanolic solution of KH-570 (2.00 g) was slowly added, and the reaction was allowed to proceed for 5 h to achieve surface grafting. Subsequently, xylene (20.00 g) was introduced, and ethanol, water, and unreacted low-molecular-weight species were removed by rotary evaporation at 40 °C, 50 rpm, and 0.09 MPa. A xylene dispersion of KH-570-modified nano-SiO_2_ with a solid content of 15 wt% was thus obtained and denoted as SiO_2_-K. The TEOS/NH_3_·H_2_O/H_2_O/ethanol composition and the subsequent KH-570 dosage were selected with reference to commonly used Stöber-type silica preparation and silane functionalization protocols reported for nanosilica-based hybrid materials [26,27], while also considering the need to obtain a stable xylene-dispersible silica intermediate with sufficient surface vinyl functionality for subsequent graft polymerization. In the present work, the formulation was therefore chosen to balance silica formation, surface modification efficiency, and dispersion processability rather than to represent a universal optimum composition.

#### 2.3.2. In Situ Graft Copolymerization of NSDA

**Figure 2:**
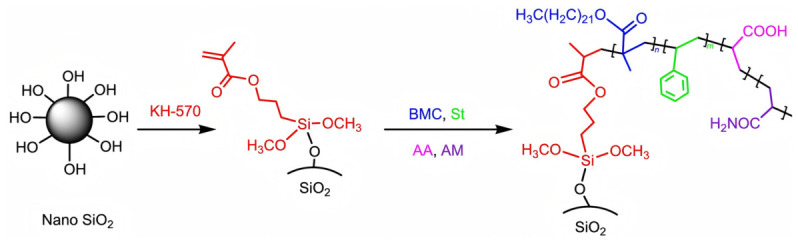
Schematic illustration of the in situ synthesis route of the NSDA nanohybrid. The colors of the structural units in the product match the text colors of their corresponding monomers.

NSDA was synthesized by in situ free-radical graft copolymerization on the surface of SiO_2_-K nanoparticles. Specifically, the SiO_2_-K dispersion containing 0.33 g of solid SiO_2_, styrene (4.60 g), BMC (4.50 g), a polar monomer mixture (0.90 g; AA/AM mass ratio = 5:4), and xylene (28.0 g) were added to a reaction flask. The system was purged with nitrogen for 30 min to remove dissolved oxygen. Subsequently, AIBN (0.06 g) dissolved in xylene was added dropwise as the initiator solution. The reaction mixture was maintained at 80 °C under continuous stirring for 6 h in an oil bath. After cooling to room temperature, the NSDA nanohybrid dispersion was obtained. The overall synthesis route is illustrated in Figure 2. The monomer feed ratio was designed according to the targeted multifunctionality of the polymer shell and literature guidance on oil-soluble heavy-oil flow improvers [16,17,18,19,29,35]. Specifically, styrene was introduced to enhance affinity for aromatic heavy fractions, BMC to provide long alkyl chains for wax-crystal regulation, AA/AM to introduce polar interaction sites. The total amount of polar monomers was kept relatively low to preserve oil-phase dispersibility, while the AA/AM mass ratio (5:4) was selected to provide a moderate balance between hydrogen-bonding capability and copolymerization compatibility. Accordingly, the present composition should be regarded as a rationally designed formulation for this study rather than a fully optimized universal recipe.

### 2.4. Characterization of NSDA

The chemical structures and functional group changes in the samples were analyzed by Fourier transform infrared spectroscopy (FT-IR, Frontier, PerkinElmer, Waltham, MA, USA) using the KBr pellet method in the range of 400–4000 cm^−1^. ^1^H NMR spectra were recorded on a 400 MHz nuclear magnetic resonance spectrometer (Bruker, Billerica, MA, USA). Thermal stability was evaluated by thermogravimetric analysis (TGA, TG 209 F1, Netzsch, Selb, Germany) under a nitrogen atmosphere at a heating rate of 10 °C/min from room temperature to 700 °C. Surface elemental composition was determined by X-ray photoelectron spectroscopy (XPS, K-Alpha, Thermo Fisher Scientific, Waltham, MA, USA). The morphology of the samples was observed by field-emission scanning electron microscopy (FE-SEM, ULTRA 55, ZEISS, Oberkochen, Germany).

### 2.5. Particle Size and Dispersity Analysis

The particle size distribution and dispersity of NSDA in xylene were evaluated by nanoparticle tracking analysis (NTA, NanoSight, Malvern Panalytical, Malvern, UK). The NSDA sample was diluted with xylene to a concentration of 0.1 wt% and transferred to the measurement cell. After equilibration at 25 °C for 5 min, the particle size distribution was recorded. Five parallel measurements were conducted, and the results were used to evaluate measurement repeatability.

### 2.6. Pour-Point Measurement

The pour point of the heavy oil before and after the addition of NSDA was determined according to the Chinese national standard GB/T 510-2018 [36]. For consistency with petroleum flow-assurance terminology in this study, the measured result is discussed here as pour point. During the measurement, the sample was cooled to the target temperature, and the sample tube was tilted at 45° for a fixed observation period to evaluate flow behavior. The lowest temperature at which the sample still exhibited flow was recorded as the pour point. Each sample was measured in duplicate, and the average value was reported.

### 2.7. Viscosity-Reduction Performance

The viscosity-reduction performance of NSDA was evaluated by measuring the apparent viscosity of heavy oil before and after treatment using a rotational viscometer [37,38] (ViscoQC 300, Anton Paar, Graz, Austria). Prior to measurement, the oil samples were preheated at 80 °C for 1 h to eliminate thermal history and ensure sample homogeneity. The samples were then transferred to the measurement cup and equilibrated at the target temperature for at least 15 min before testing. Unless otherwise specified, viscosity measurements for dosage-dependent evaluation were carried out at 50 °C. All apparent viscosity values reported in this work were measured using the SC4-21 measuring system under the same fixed testing condition, corresponding to a shear rate of 0.93 s^−1^, in order to ensure direct comparability among samples with different NSDA dosages. Since heavy oil may exhibit shear-dependent flow behavior, the present data should be interpreted as apparent viscosities measured under this fixed condition rather than as a complete rheological characterization. The viscosity reduction rate ($\eta_{VRR}$) was calculated according to Equation (1):

(1) $$\eta_{VRR}\;=\;\frac{\mu_{0}\;-\;\mu_{1}}{\mu_{0}}\;\text{×}\;100\%$$

where $\mu_{0}$ and $\mu_{1}$ represent the dynamic viscosity (mPa·s) of the original heavy oil and the oil treated with NSDA, respectively. Each measurement was performed in triplicate, and the average values were recorded to minimize experimental error.

## 3. Results and Discussion

### 3.1. Morphology and Dispersion Behavior of NSDA Hybrid

To elucidate the impact of surface modification on the physical characteristics of the nano-SiO_2_-K core and its subsequent behavior as a flow improver, the morphology and oil-phase dispersion of NSDA were investigated using scanning electron microscopy (SEM) and nanoparticle tracking analysis (NTA). It should be noted that SEM reflects the dried-state morphology after solvent removal, whereas the dispersion behavior in an oil-like medium is more appropriately evaluated by NTA in xylene. The results suggest that the “grafting-from” polymerization strategy transforms the aggregated SiO_2_-K precursor into nanoscale NSDA hybrids with improved dispersion in xylene, which is potentially beneficial for flow-improvement performance.

**Figure 3:**
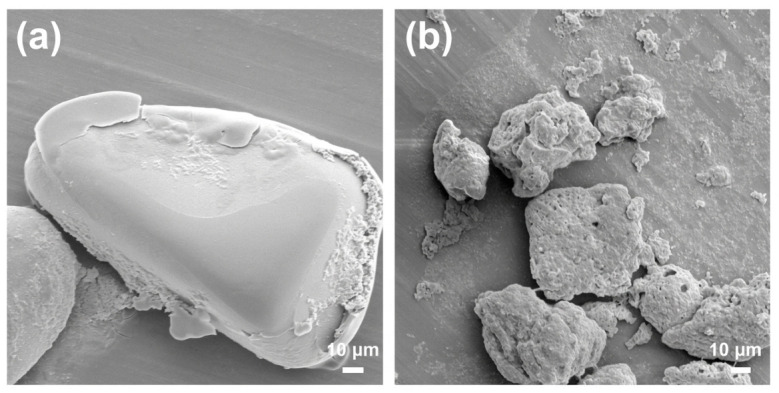
SEM images of (**a**) SiO_2_-K and (**b**) NSDA.

The micromorphology and structural evolution from SiO_2_-K to NSDA were first examined by SEM, as shown in Figure 3. For the precursor SiO_2_-K (Figure 3a), the particles exhibit a large, dense, and relatively smooth block-like morphology, indicative of strong self-association of primary silica particles. This suggests that while KH-570 modification alters the surface characteristics, it is insufficient to fully suppress the intrinsic tendency of nano-silica to form compact aggregates in solid state. In stark contrast, NSDA (Figure 3b) displays a distinctly different morphology, characterized by irregular fragmented aggregates with a rough and porous surface. The large compact aggregates observed for SiO_2_-K are no longer evident; instead, smaller and more discrete domains are distributed within the three-dimensional network formed by the multicomponent copolymer containing BMC, St, AA, and AM. This morphological evolution provides visual evidence for the successful incorporation of silica cores into the polymer matrix during the in situ graft-copolymerization process. The significant change from large, dense blocks to smaller, fragmented structures highlights the steric effects of the grafted polymer chains in disrupting strong inter-particle attractions. It is worth noting that while SEM visualizes the aggregated state of NSDA upon solvent evaporation, such dried-state images cannot by themselves represent the actual colloidal state in oil or aromatic solvent; therefore, the dispersion behavior discussed below is based primarily on NTA measurements in xylene.

**Figure 4:**
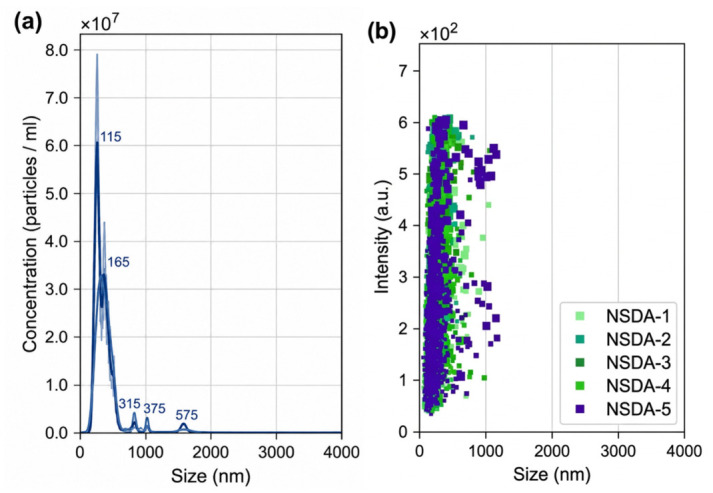
NTA results of NSDA: (**a**) particle size distribution and (**b**) size-intensity scatter plots (n = 5).

To further evaluate the oil-phase dispersion behavior and nanoscale dimensional characteristics of NSDA, the sample was dispersed in xylene (0.1 wt%) and analyzed by nanoparticle tracking analysis (NTA). As shown in Figure 4a, NSDA exhibited a narrow nanoscale distribution with a dominant peak at approximately 115 nm and a weaker secondary feature around 165 nm, indicating excellent dispersion uniformity in the aromatic solvent without obvious macroscopic flocculation. Statistically, the particle population shows a mode diameter of 115 nm, with most particles remaining within the nanoscale range. The slightly right-skewed distribution, including minor populations at 315, 375, and 575 nm, may be attributed to a small fraction of larger entities arising from limited secondary association, such as polymer-chain entanglement or weak clustering of silica-containing domains. Although no obvious macroscopic flocculation was observed under the present dispersion and testing conditions, these larger populations suggest that the colloidal state is not entirely monodisperse. Their possible influence on long-term storage stability or stability under prolonged flow conditions warrants further investigation. To assess the reliability of the measurements, particle size-intensity scatter plots were obtained from five parallel runs of the same dispersion. As shown in Figure 4b, the five datasets (NSDA-1 to NSDA-5) overlap closely and exhibit a highly consistent intensity-size relationship, confirming good repeatability of the measurements and the absence of unstable macroscopic flocculates.

The observed predominantly nanoscale dispersion and specific morphological features of NSDA hybrid particles are considered important for their proposed dual-functional performance. The “grafting-from” strategy effectively provides sufficient steric stabilization to suppress large-scale agglomeration in an oil-like medium. This excellent dispersibility, together with the nanoscale dimensions and accessible hybrid surface of the particles, is expected to facilitate rapid adsorption at interfaces and effective interaction of the amphiphilic polymer segments with crude oil components. Specifically, it is hypothesized that this uniform nanoscale structure will enable more effective wax crystal regulation, disruption of asphaltene and resin associations, and a “micro-bearing” effect, thereby contributing significantly to the observed viscosity reduction and pour point depression. Although a direct side-by-side dispersion comparison with pristine SiO_2_ was not included in the present work, the combined SEM, NTA, and surface-composition results consistently suggest that the KH-570-assisted grafting strategy effectively reduces the tendency of silica-containing domains to form large compact aggregates and improves their dispersibility in an oil-like aromatic medium. A more rigorous quantitative comparison with unfunctionalized silica will be valuable in future studies.

Within the time window of the NTA measurements, no evident macroscopic sedimentation or unstable flocculation was observed for the diluted NSDA dispersion in xylene, suggesting acceptable short-term dispersion stability under the test conditions. However, longer-term storage stability and temperature-dependent re-agglomeration behavior were not systematically investigated in the present work and therefore warrant further study.

### 3.2. Surface Structural Composition of NSDA Hybrid

To verify the successful construction of the organic-inorganic hybrid interface and correlate the chemical nature with the observed morphology changes (Section 3.1), a combination of FT-IR, ^1^H NMR, TGA, and XPS was employed. These results provide conclusive evidence for the covalent grafting of the multifunctional copolymer onto the nano-silica surface.

**Figure 5:**
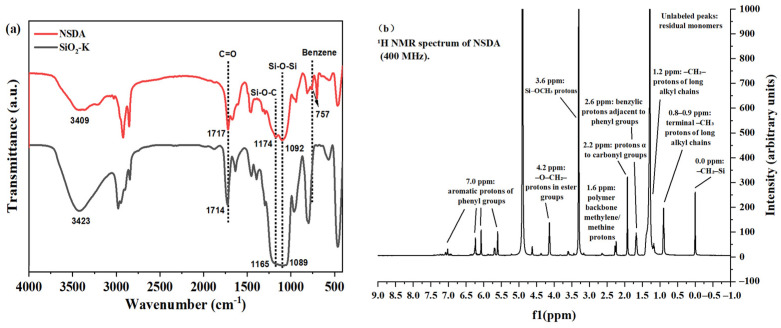
Structural characterization of NSDA: (**a**) FT-IR spectra of SiO_2_-K and NSDA; (**b**) ^1^H NMR spectrum of NSDA.

The chemical functional groups of the samples were first characterized by FT-IR and ^1^H NMR (Figure 5). As shown in Figure 5a, the FT-IR spectrum of NSDA exhibits significant changes compared to the precursor SiO_2_-K. The broad hydroxyl stretching band at 3409 cm^−1^ in NSDA is noticeably weaker than that of SiO_2_-K (3423 cm^−1^), indicating the partial consumption of surface silanol groups (Si-OH) via condensation with KH-570. The emergence of a prominent carbonyl stretching vibration (C=O) at 1717 cm^−1^ confirms the successful incorporation of ester groups from the polymer segments. Meanwhile, the characteristic intense asymmetric stretching vibration of Si-O-Si at 1092 cm^−1^, accompanied by the symmetric stretching at lower frequencies, and the enhanced C-O stretching at 1174 cm^−1^ further support the formation of a covalently anchored polymer shell. Additionally, the absorption at 757 cm^−1^ (styrenic benzene ring) and the intensified signals in the 2850–2950 cm^−1^ region (aliphatic C-H stretching) demonstrate that the SiO_2_ surface is chemically integrated with multifunctional organic moieties rather than being merely physically coated. This robust chemical bonding likely contributes to the suppressed secondary aggregation and improved nanoscale dispersion observed in the previous morphological analysis.

Further structural information was obtained from the ^1^H NMR spectrum of NSDA (Figure 5b). It should be noted that this spectrum is interpreted here as supportive evidence for the organic shell composition, rather than as a stand-alone proof of a fully resolved grafted structure on the silica surface. The resonance near 0.0 ppm can be assigned to methylene protons adjacent to silicon (-CH_2_-Si), which is consistent with the presence of the silane-derived interfacial bridge. The signals at 0.8–0.9 ppm and 1.2 ppm are attributable to the terminal methyl (-CH_3_) and internal methylene (-CH_2_-) protons of the long alkyl chains, respectively, while the signal around 7.0 ppm is consistent with aromatic units. The resonances at 2.2 ppm (-CH_2_-C(=O)-) and 4.2 ppm (-O-CH_2_-) further support the ester-containing copolymer structure. Taken together with the FT-IR, TGA, and XPS results, the ^1^H NMR data support the successful formation of an organic shell associated with the silica-based hybrid architecture.

**Figure 6:**
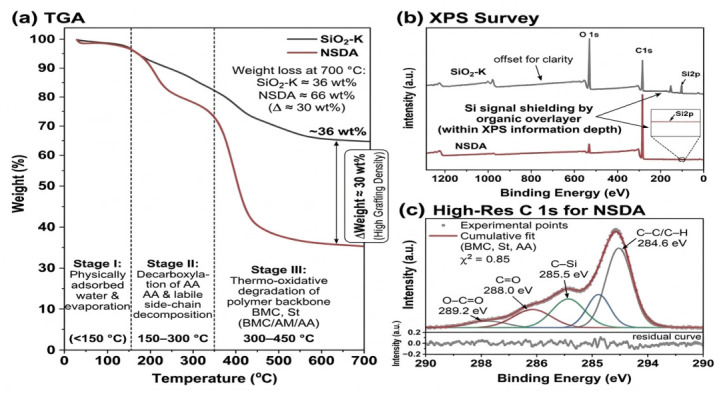
TGA and XPS characterization of SiO_2_-K and NSDA: (**a**) TGA curves and weight loss at 700 °C; (**b**) XPS survey spectra (offset for clarity) showing attenuation of the Si 2p signal for NSDA; (**c**) high-resolution C 1s spectrum of NSDA with peak deconvolution.

The thermal stability and quantitative grafting efficiency were evaluated by TGA (Figure 6a). NSDA exhibits a characteristic three-step mass-loss profile. Following the initial removal of adsorbed moisture below 150 °C, the second stage (150–300 °C) is attributed to the decarboxylation of acrylic acid units. The primary mass loss occurring between 300 °C and 450 °C corresponds to the thermo-oxidative degradation of the grafted polymer backbone. Notably, the total weight loss of NSDA reaches 66 wt%, which is 30 wt% higher than that of SiO_2_-K, This difference provides a semi-quantitative estimate of the additional grafted organic fraction associated with the in situ copolymer shell, supporting a relatively high degree of surface grafting in NSDA. Compared to pure polymer additives, NSDA demonstrates superior high-temperature integrity, as the inorganic SiO_2_ core acts as a physical barrier to the diffusion of volatile degradation products [39]. This enhanced thermal robustness suggests that the NSDA hybrid is better able to maintain its structural integrity without premature degradation during high-temperature reservoir injection, which is vital for sustained flow-improvement performance under the harsh thermal conditions of heavy-oil production.

Finally, the surface elemental composition was confirmed by XPS (Figure 6b,c). The survey spectrum of NSDA shows a dramatic increase in the C 1s signal accompanied by a significant attenuation of the Si 2p peak, indicating that the silica core is effectively encapsulated by a thick organic overlayer. High-resolution C 1s deconvolution (Figure 6c) identifies five distinct chemical states: C-C/C-H (284.6 eV), C-Si (285.5 eV), C-O (286.3 eV), C=O (288.0 eV), and O-C=O (289.2 eV). These results are in excellent agreement with the monomeric units (BMC, St, AA, and AM) identified by FT-IR and NMR. In conclusion, the multi-method characterization confirms the successful construction of a covalently integrated organic-inorganic hybrid architecture. The chemically diverse surface of NSDA, featuring both polar and non-polar moieties, is expected to play an important role in regulating interfacial interactions with waxes and asphaltenes [40].

### 3.3. Pour Point and Viscosity-Reduction Performance

**Figure 7:**
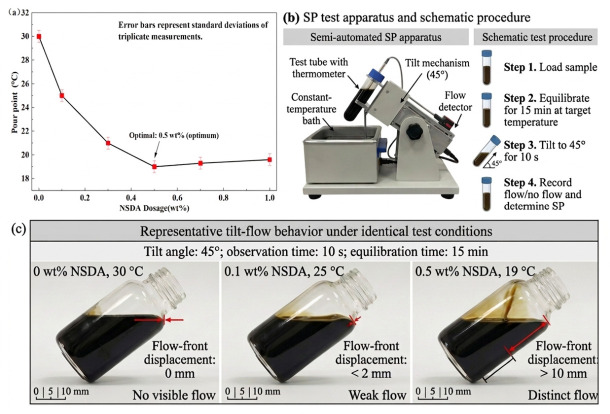
Effect of NSDA dosage on the pour point and flow behavior of Liaohe heavy oil. (**a**) Pour point of Liaohe heavy oil as a function of NSDA dosage. (**b**) Semi-automated SP test apparatus and schematic procedure. (**c**) Representative tilt-flow behavior under identical test conditions.

Following the structural validation of the NSDA hybrid, its practical efficacy in regulating the rheological properties of Liaohe heavy oil was systematically investigated. The pour point (PP) was first evaluated as a critical parameter reflecting the low-temperature mobility of the crude oil, with results summarized in Figure 7. As shown in Figure 7a, the untreated heavy oil exhibited an initial PP of 30 °C, which decreased significantly upon the addition of NSDA in a dosage-dependent manner. The PP dropped to 25 °C at 0.1 wt% NSDA and reached a minimum value of 19 °C at an optimal dosage of 0.5 wt%. Notably, further increasing the dosage to 0.7 wt% or 1.0 wt% resulted in a plateau or a slight rebound in the PP (20 °C), suggesting that 0.5 wt% may be close to an effective dosage threshold under the present experimental conditions. The reduced incremental benefit at higher dosages may be related to less efficient additive distribution and/or limited secondary association of a fraction of the hybrid particles, which could weaken their cooperative interaction with wax components; however, this interpretation should be regarded as tentative in the absence of direct in-oil microscopic evidence [28,35,41,42]. These quantitative findings are further supported by the visual flow-behavior observations in Figure 7c. Under identical test conditions, the untreated oil remained immobile at 30 °C, whereas the sample treated with 0.5 wt% NSDA displayed distinct and continuous flow even at 19 °C, with a flow-front displacement exceeding 10 mm. This stark contrast demonstrates that NSDA can effectively disrupt the internal structural resistance of Liaohe heavy oil at low temperatures.

In this study, the monomer formulation was selected to provide a practical balance among aromatic affinity, wax-crystal regulation, and polar interaction capability. A systematic optimization of monomer-feed ratio was beyond the main scope of the present work, although it would be valuable for future composition-property studies.

**Figure 8:**
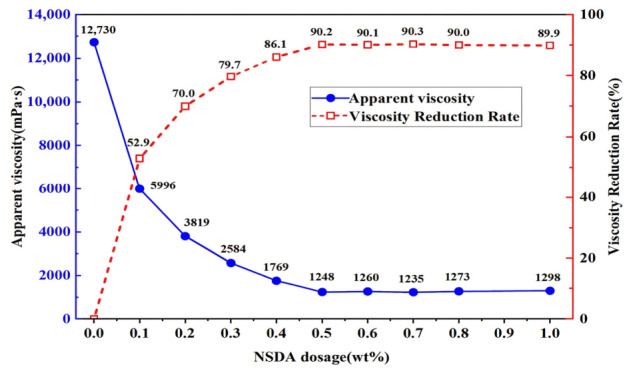
Effect of NSDA dosage on the apparent viscosity and viscosity reduction rate of Liaohe heavy oil at 50 °C.

The viscosity-reduction performance of NSDA at 50 °C, evaluated as apparent viscosity under a fixed shear condition, further corroborates its potential as a multifunctional flow improver, as illustrated in Figure 8 and Table 3. The untreated Liaohe heavy oil possessed a high apparent viscosity of 12,730 mPa·s, indicating a densely associated internal microstructure. Upon the introduction of NSDA, the apparent viscosity underwent a dramatic decline, particularly within the 0.1–0.5 wt% range. At the optimal dosage of 0.5 wt%, the viscosity plummeted to 1248 mPa·s, achieving a remarkable viscosity reduction rate of 90.2%. Consistent with the pour-point trends, the viscosity remained nearly constant between 0.5 wt% and 1.0 wt%, with a maximum reduction rate of 90.3% recorded at 0.7 wt%. These results imply that the interfacial activity and dispersion effects of the hybrid particles reach an effective equilibrium at a relatively low concentration, beyond which additional NSDA contributes marginally to further structural disruption [43].

Compared with representative oil-soluble polymeric reducers, copolymer-based additives, and nanoparticle-assisted systems reported in the literature [3,15,16,17,18,23,44], the NSDA hybrid developed in this work exhibits competitive viscosity-reduction capability at relatively low dosage while simultaneously providing measurable pour-point depression. Although direct one-to-one quantitative comparison is not straightforward because of differences in crude-oil source, additive dosage, test temperature, and rheological protocol among studies, the present results nonetheless demonstrate the effectiveness of integrating multifunctional polymer segments with a rigid nanosilica core for synergistic heavy-oil flow improvement.

The viscosity data reported here were obtained under a fixed measurement condition and are mainly intended for comparative evaluation of additive performance. A broader rheological assessment over variable shear rates would be valuable in future work to better reflect practical pipeline-flow conditions.

The pronounced dual-functional performance of NSDA can be reasonably attributed to the synergistic interplay between its multifunctional polymer shell and the rigid nano-SiO_2_ core. For low-temperature flow behavior and pour-point depression, the long alkyl chains derived from BMC are expected to play an important role by interacting with paraffinic components and disturbing the regular growth and interlocking of wax crystals during cooling. In parallel, the aromatic styrenic units may interact with aromatic heavy fractions and help regulate the aggregation state of asphaltene-rich species, while the polar functional groups introduced by AA and AM can interfere with hydrogen bonding and other polar associations among resins and asphaltenes, thereby facilitating dispersion of heavy components [44].

It should be noted, however, that the apparent viscosity measurements in this study were conducted at 50 °C. Therefore, the viscosity reduction observed at this temperature is more reasonably attributed mainly to the weakening of resin/asphaltene-associated microstructures, improved dispersion of heavy fractions, and the physical spacer/lubrication contribution of the nanoscale hybrid particles, rather than to direct regulation of wax crystals. In this context, the inorganic nano-SiO_2_ core may contribute a nanoscale “micro-bearing” or spacing effect by reducing direct frictional contact among associated microdomains and facilitating their relative movement under shear. Similar nanoscale physical contributions have been discussed in previous reports on nanoparticle-assisted viscosity reduction and flow improvement in heavy oil systems [18,22,23,24,40]. Unlike conventional pure polymer additives, NSDA combines this nanoscale physical contribution with the multi-site chemical regulation provided by the grafted shell. The nanoscale dimensions of the hybrid architecture may also improve accessibility to complex local environments in heavy oil, enabling the functional segments to participate more effectively in interfacial regulation. Accordingly, wax-related regulation is considered more relevant to the observed pour-point depression, whereas the viscosity reduction at 50 °C is interpreted primarily in terms of heavy-component association weakening and dispersion enhancement.

### 3.4. Proposed Synergistic Mechanism for Viscosity Reduction and Pour-Point Depression

**Figure 9:**
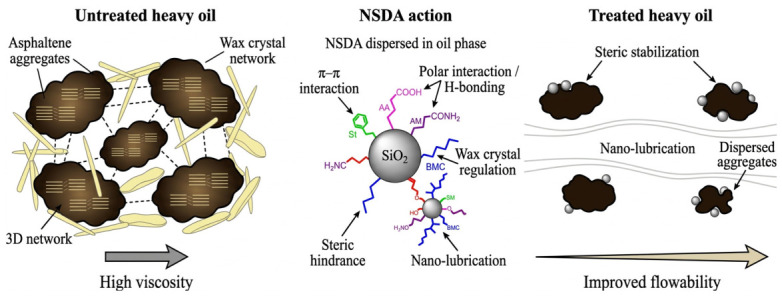
Proposed schematic illustration of the synergistic mechanism of NSDA for viscosity reduction and pour-point depression in heavy oil.

Based on the morphology and surface structural characterization of the NSDA hybrid presented in Section 3.1 and Section 3.2, together with its pour-point-depression and viscosity-reduction performance in Section 3.3, a plausible synergistic mechanism is proposed to explain its dual flow-improvement function in Liaohe heavy oil, as schematically illustrated in Figure 9.

In the absence of NSDA, Liaohe heavy oil may be regarded as a highly associated multiphase microstructure involving asphaltene-rich aggregates, polar heavy components, and, under cooling conditions, interlocking wax-crystal structures (Figure 9, left). This complex microstructure is sustained by multiple intermolecular interactions, including π–π association among aromatic asphaltene cores, hydrogen bonding and other polar interactions among resinous and asphaltenic species, as well as the physical interlocking of precipitated wax crystals, particularly at lower temperatures [45]. The coexistence of these interactions gives rise to strong internal structural resistance, which is responsible for the high apparent viscosity and poor low-temperature flowability of the untreated heavy oil [46].

After the addition of NSDA, the hybrid nanoparticles become dispersed in the oil phase and progressively weaken the original associated network through the cooperative action of the functional polymer shell and the rigid inorganic core (Figure 9, center). On the one hand, the polymer shell provides multiple interaction sites for heavy-oil components. The aromatic segments can associate with asphaltenes and weaken their tendency to form large aggregates, while the polar groups derived from AA and AM can interfere with the original polar association network within the oil phase. Meanwhile, the long alkyl side chains derived from BMC exhibit good affinity for paraffinic components and contribute to wax-crystal regulation by disturbing the ordered growth and interlocking of wax crystals during cooling [11,47]. These combined molecular-level interactions are consistent with the marked reduction in pour point and viscosity observed in Section 3.3. However, the contribution of wax-crystal regulation is expected to be more relevant to low-temperature flow behavior, whereas the viscosity reduction measured at 50 °C is more closely associated with changes in heavy-component association and dispersion.

On the other hand, the nano-SiO_2_ core provides additional physical regulation beyond the chemical functionality of the polymer shell. As the associated structure is weakened, the heavy-oil system tends to evolve into smaller and more dispersed microdomains (Figure 9, right). The grafted polymer layer on the particle surface contributes to steric stabilization, which helps suppress the re-association of these dissociated domains. At the same time, the rigid nano-SiO_2_ core may act as a nanoscale spacer and contribute to a nano-lubrication effect (i.e., a micro-bearing effect), thereby reducing direct sliding friction between adjacent aggregates and facilitating their relative movement under shear stress [22,43]. Owing to the nanoscale dimensions of the hybrid particles, the functional shell segments can more effectively access the complex micro-environment within heavy oil and participate in interfacial and molecular-level regulation.

Overall, the excellent flow-improvement performance of NSDA may be attributed to a multiscale synergistic mechanism involving aggregate dissociation, disruption of polar associations, wax-crystal regulation, steric stabilization, and nanoparticle-assisted nano-lubrication. Through the cooperative integration of these effects, NSDA is proposed to weaken the interconnected microstructure of heavy oil, thereby enabling simultaneous viscosity reduction and pour-point depression at relatively low dosage [48,49,50].

## 4. Conclusions

In this study, a multifunctional organic–inorganic nanohybrid (NSDA) was successfully synthesized via in situ free-radical copolymerization on KH-570-modified nano-SiO_2_ surfaces. The comprehensive characterization confirmed the covalent grafting of the polymer shell, which endowed the hybrid with superior thermal stability and oil-phase dispersibility. The key findings are summarized as follows:

1. Exceptional Dual-Function Performance: At a dosage of 0.5 wt%, the NSDA hybrid achieved a viscosity reduction rate of 90.2% and a pour-point depression of 11 °C in Liaohe heavy oil, demonstrating its high efficiency as both a viscosity reducer and a pour-point depressant.
2. Multiscale Synergistic Mechanism: The flow-improving capability is attributed to the synergy between the organic shell and the inorganic core. The polar and aromatic groups disrupt the asphaltene/resin associative networks, the long alkyl chains regulate wax crystallization, and the rigid nano-SiO_2_ provides a nanoscale “micro-bearing” effect.
3. Stability and Practicality: The robust covalent bonding between the polymer and SiO_2_ is expected to contribute to stable performance under elevated-temperature conditions, making NSDA a promising candidate for heavy-oil flow assurance in complex environments.

Overall, this research provides a robust theoretical and empirical foundation for developing advanced nanohybrids for heavy oil viscosity reduction. Future work will systematically examine the long-term dispersion stability, temperature-dependent adaptability, and salinity tolerance of the NSDA hybrid under specific reservoir and transportation conditions. Moreover, inspired by the recent successful applications of functional polymer microspheres and robust particle gels in harsh reservoir environments [51,52], further studies will also focus on assessing the synergy of this nanohybrid with other advanced enhanced oil recovery (EOR) technologies.

## Figures and Tables

**Table 1:** Main physicochemical information of the reagents used in this study.

Reagent	Role in Synthesis	Purity/Grade	Representative Physicochemical Information *
Styrene (St)	Aromatic monomer	AR	C_8_H_8_; Mw 104.15 g/mol
Docosyl methacrylate (BMC)	Long-chain alkyl monomer	AR	C_26_H_50_O_2_; Mw 394.67 g/mol
Acrylic acid (AA)	Polar monomer	AR	C_3_H_4_O_2_; Mw 72.06 g/mol
Acrylamide (AM)	Polar monomer	AR	C_3_H_5_NO; Mw 71.08 g/mol
3-(trimethoxysilyl)propyl methacrylate (KH-570)	Silane coupling agent	AR	C_10_H_20_O_5_Si; Mw 248.35 g/mol
Tetraethyl orthosilicate (TEOS)	Silica precursor	AR	C_8_H_20_O_4_Si; Mw 208.33 g/mol
AIBN	Radical initiator	≥98%	C_8_H_12_N_4_; Mw 164.21 g/mol
Xylene	Dispersion/reaction solvent	AR	Mixed xylene isomers
Ethanol	Co-solvent	AR	C_2_H_5_OH; Mw 46.07 g/mol
Aqueous ammonia	Catalyst	ACS	NH_3_·H_2_O

* Information summarized from the supplier’s technical data sheets.

**Table 2:** Basic physicochemical properties of the crude oil.

Parameter	Value
Density, g/cm^3^, at 20 °C	0.955
Viscosity, mPa·s, at 50 °C	12,730
Pour point, °C	30
Wax content, wt%	8.0
Saturates, wt%	29.56
Aromatics, wt%	37.49
Resins, wt%	13.92
Asphaltenes, wt%	19.03

**Table 3:** Apparent viscosity and viscosity reduction rate of Liaohe heavy oil at 50°C as a function of NSDA dosage.

NSDA Dosage (wt%)	Apparent Viscosity (mPa·s)	Viscosity Reduction Rate (%)
0.0	12,730	0.0
0.1	5996	52.9
0.2	3819	70.0
0.3	2584	79.7
0.4	1769	86.1
0.5	1248	90.2
0.6	1260	90.1
0.7	1235	90.3
0.8	1273	90.0
1.0	1298	89.9

## Data Availability

The original contributions presented in this study are included in the article. Further inquiries can be directed to the corresponding authors.
